# Hydrocarbon pneumonitis caused by the inhalation of wood preservative

**DOI:** 10.1002/rcr2.379

**Published:** 2018-10-26

**Authors:** Munechika Hara, Shin‐ichiro Iwakami, Issei Sumiyoshi, Takashi Yoshida, Shinichi Sasaki, Kazuhisa Takahashi

**Affiliations:** ^1^ Department of Respiratory Medicine Juntendo University Shizuoka Hospital Izunokuni Shizuoka Japan; ^2^ Department of Respiratory Medicine Juntendo University Graduate School of Medicine Bunkyo‐Ku Tokyo Japan; ^3^ Department of Respiratory Medicine Juntendo University Urayasu Hospital Urayasu Chiba Japan

**Keywords:** Corticosteroid, hydrocarbon pneumonitis, kerosene, respiratory failure, wood preservative

## Abstract

A 69‐year‐old man was admitted to our hospital with acute dyspnoea, which developed after using a wood‐preserving agent in an enclosed space. Burn injuries were evident on his face, neck, chest, and both upper arms. Bronchoalveolar lavage was carried out. The collected fluid resembled wood preservative. Subsequently, it was established that kerosene was a major component of the wood preservative. A diagnosis of hydrocarbon pneumonitis was established. The patient’s respiratory and general findings improved with intensive care, which included mechanical ventilation. Corticosteroid was not required to aid his recovery. Aspiration and/or inhalation of hydrocarbon compounds, such as kerosene, turpentine, and gasoline, can cause acute and fatal pneumonitis. In managing cases of hydrocarbon pneumonitis, a prompt diagnosis and appropriate supportive care are important to achieve a good outcome.

## Introduction

Aspirating and/or inhaling hydrocarbon products can cause acute and fatal pneumonitis 1. This report focuses on a case of hydrocarbon pneumonitis caused by inhaling wood preservative in an enclosed space and demonstrates successful care without the use of corticosteroids.

## Case Report

**Figure 1:**
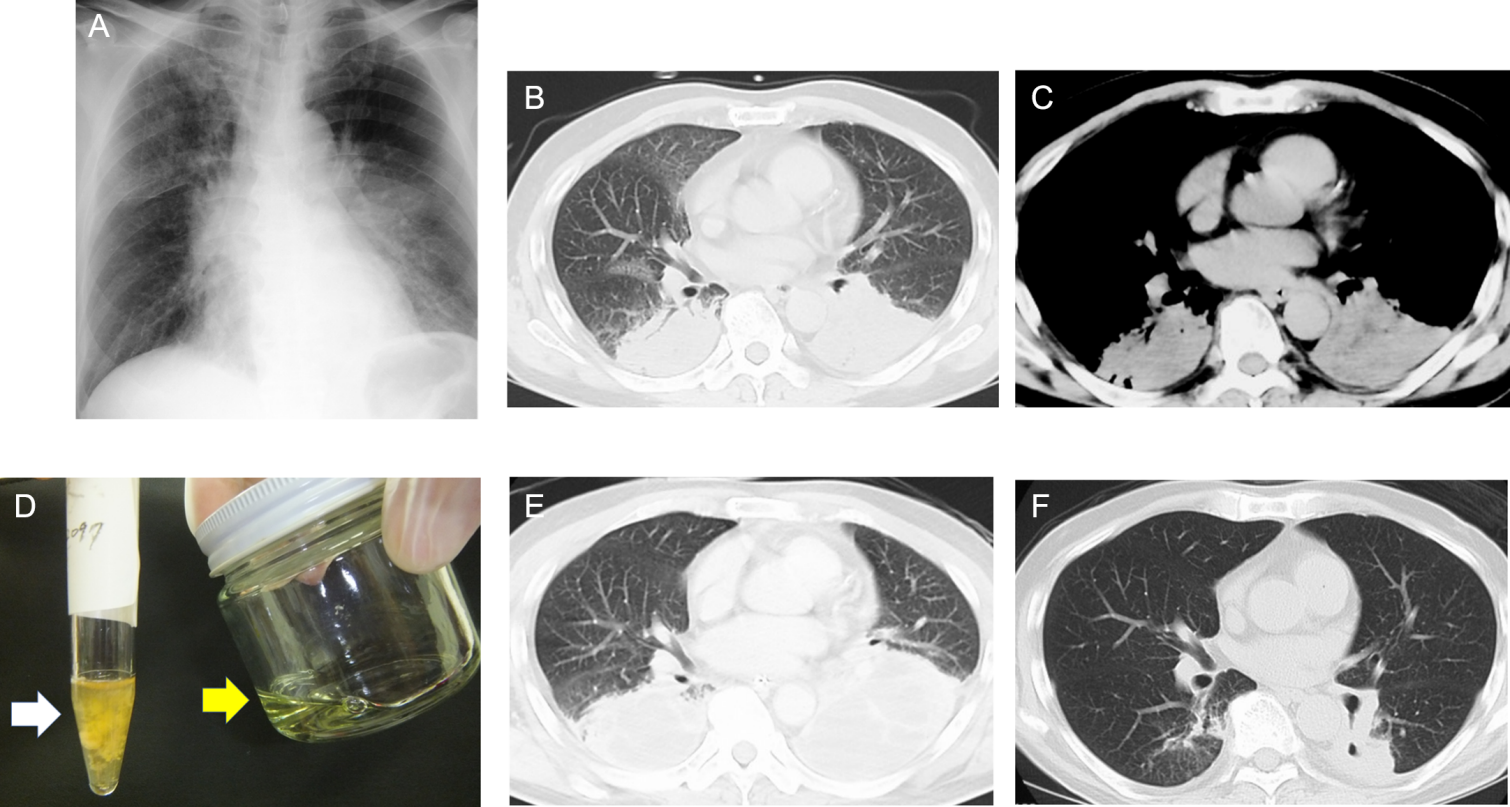
(A) Chest X‐ray showing the increment of bilateral hilar shadow. (B) Thoracic computed tomography (CT) showing infiltrative shadows in both lower lobes. (C) Thoracic CT showing low‐density area in infiltrative shadows in both lower lung lobes using a mediastinal window. (D) The collected fluid using bronchoalveolar lavage (white arrow) resembling wood preservative (yellow arrow). (E) Thoracic CT at 2 weeks after the first CT, showing worsening of infiltration of both lower lobes. (F) Radiological improvement was noted a month after the first CT.

A 69‐year‐old man was referred to our hospital because he developed dyspnoea and delirium two days after working with wood preservative in an enclosed space. He had been exposed to the agent for about eight hours on each of the two days. He had a current 60 pack‐year smoking history. On presentation, he was tachycardic (pulse rate of 88/min) and tachypnoeic (respiration rate of 20/min), with laboured breathing. Oxygen saturation was 90% on 10 L/min oxygen via a reservoir mask. Burn injuries were apparent on his face, neck, chest, and both upper arms. Bibasal coarse crackles were present on chest auscultation. Brain computed tomography (CT) demonstrated no remarkable phenomena. He had an elevated white cell count with 90% neutrophils, and the C‐reactive protein (CRP) was 23.8 mg/dL. Arterial blood gas examination results were as follows: pH 7.40, PaCO_2_ 31.8 mmHg, PaO_2_ 63.7 mmHg, and HCO_3_^−^ 20 mmol/dL (10 L/min oxygen via a reservoir mask). The chest X‐ray on admission demonstrated increased bilateral hilar shadows (Fig. 1A), and thoracic CT indicated infiltration in both lower lobes (Fig. 1B), which was observed to have a low‐density area in the infiltrative shadow using mediastinal windows (Fig. 1C). His ECG was 82 bpm in the normal range, and no cardiac enzyme was elevated. Because burning of the respiratory tract was expected in connection with the burn injury on his face and neck, intratracheal intubation and mechanical ventilation were performed in anticipation. Subsequently, bronchoalveolar lavage (BAL) was carried out, and the return fluid (Fig. 1D, white arrow) resembled the wood preservative (Fig. 1D, yellow arrow). The BAL differential count demonstrated a neutrophilia of 46% (the normal range; 3% or less). Bacteriological culture and acid‐fast bacilli stains were negative. Kerosene was assessed to be the main component of the wood preservative. The diagnosis of hydrocarbon pneumonitis caused by inhalation of this agent containing kerosene was established based on these findings. Antimicrobials were administered because of potential infectious complications. In addition, intensive treatment, including mechanical ventilation and hydration, was provided. Although the infiltration of both lower lung fields worsened temporarily (Fig. 1E), the patient’s general condition and radiological findings gradually improved with persistent, intensive management (Fig. 1F). He was successfully weaned off mechanical ventilation and was discharged after two months. He fully recovered without any residual deficits after his discharge.

## Discussion

Hydrocarbons are organic compounds, categorized as aliphatic and aromatic 1. Three major types of hydrocarbon exposure have been described: (1) children who accidentally ingest household hydrocarbons; (2) workers who are exposed via transdermal or inhalational routes; and (3) adolescents or young adults who intentionally inhale 1. Acute hydrocarbon exposure can cause multiple organ dysfunction, and patients can present with encephalopathy, pneumonitis, arrhythmia, acidosis, and dermatitis 1.

Hydrocarbon pneumonitis is an acute pneumonitis caused by aspiration and/or inhalation of hydrocarbon compounds with low viscosity and high volatility, such as kerosene, turpentine, and gasoline, most of which are members of the paraffin, naphthene, or aromatic classes 2. Hydrocarbons disrupt surfactants, resulting in the decrease of pulmonary compliance 1. They additionally cause pulmonary injury, with resultant inflammation, oedema, and necrosis 1. The clinical presentation of hydrocarbon pneumonitis is often non‐specific and includes breathlessness, cough, chest pain, and haemoptysis 3. Hydrocarbon pneumonitis usually improves over a few days with supportive measures. However, cases of severe cavitatory pneumonia and acute respiratory distress syndrome have also been described 2.

In this case, the patient stated that he used wood preservatives in an enclosed space. It is likely that he inhaled volatized hydrocarbons during his work. As a result, he developed encephalopathy, pneumonitis, and dermatitis due to being exposed to the wood preservative containing hydrocarbons, with the additional development of acute respiratory distress syndrome.

Typical findings on chest radiography of hydrocarbon pneumonitis consist of bilateral infiltrates, usually in the middle and lower lobes. Radiological abnormalities can persist for several months 1. The chest radiological findings of the present case demonstrated infiltration in both lower lobes, and the findings persisted for about two months. The low‐density area in the infiltrate, using mediastinal windows, was considered a necrotic area caused by marked inflammation.

Supportive care, including mechanical ventilation, is the main treatment for hydrocarbon pneumonitis 1. There is no good supporting evidence for the use of either corticosteroids or antimicrobials 1, 2. Whilst corticosteroids have been described to be beneficial in some cases 4, the same effects were not seen in others 5. Antimicrobials were administered in this case because of the possibility of infectious complications, and the patient improved without corticosteroids. This case demonstrates that prompt diagnosis and appropriate supportive care are vital in the management of hydrocarbon pneumonitis and can be associated with a good outcome.

### Disclosure Statement

Appropriate written informed consent was obtained for publication of this case report and accompanying images.
